# Simultaneous extraction of mRNA and microRNA from whole blood stabilized in tempus tubes

**DOI:** 10.1186/s13104-019-4087-5

**Published:** 2019-01-18

**Authors:** Jendai Richards, Elizabeth R. Unger, Mangalathu S. Rajeevan

**Affiliations:** Division of High-Consequence Pathogens and Pathology, Centers for Disease Control and Prevention, 1600 Clifton Road, Atlanta, GA 30329 USA

**Keywords:** Stabilized whole blood, RNA extraction, mRNA and miRNA representation, Tempus tubes, Biobanks

## Abstract

**Objective:**

Studies of mRNA and miRNA expression profiling increasingly use stabilized whole blood. Commercial RNA extraction kits do not provide information about the simultaneous recovery of both mRNA and miRNA. This study evaluated yield, quality, integrity and representation of mRNA and miRNA from whole blood stabilized in Tempus tubes using three RNA extraction kits; two filter-based (Tempus and Norgen) and one bead-based (MagMax; manual vs. semi-automated, and with and without DNase treatment).

**Results:**

All RNA extraction kits and methods resulted in similar yields of mRNA (total RNA yield, quality, integrity and representation) whereas there were differences in yields of miRNA. MagMax, either manual or semi-automated, with or without DNase treatment, yielded 1.6–2.2-fold more miRNA than Tempus and Norgen kits. In addition, MagMax and Norgen methods gave greater than 12-fold more and 3.3-fold less enrichment of specific miRNA targets, respectively, in comparison to Tempus extraction reagents. This study identified MagMax kit for simultaneous recovery of both mRNA and miRNA from whole blood collected in Tempus tubes.

## Introduction

Gene expression profiling is only meaningful when the starting RNA is representative of the starting material. Representation and yield of RNA are impacted by a number of pre-analytic factors including RNA extraction [1]. While immediate extraction of fresh blood can minimize ex vivo instability of RNA, this is not a realistic option in many settings and is subject to variations due to timing and storage conditions. Collection methods that immediately lyse cells chemically and stabilize nucleic acids, such as PAXgene (Qiagen Inc, Germantown, MD) and Tempus Blood RNA (Thermo Fisher, Waltham, MA) are increasingly used, thus simplifying storage and collection and allowing batched extraction. The manufacturers of these blood stabilizing systems supply standard RNA extraction reagents (PAXgene Blood RNA kit, Qiagen and Tempus Spin RNA Isolation kit, Thermo Fisher) for recovery of good quality RNA for messenger RNA (mRNA) profiling [2]. However, these kits are not recommended for extraction of microRNA (miRNA), i.e., RNA that is 18–24 nucleotide in length, as RNAs less than 200 nucleotides are not recovered in the final extract [2–4]. With increasing interest in studying the expression pattern of miRNAs, an extraction method that simultaneously recovers both mRNA and miRNA is highly desirable. More recently, new RNA extraction kits, such as Norgen Preserved Blood RNA purification kit I (Norgen Biotek Corp, Ontario, Canada) and MagMax for stabilized blood tubes RNA isolation kit (Thermo Fisher) have been developed for use with stabilized whole blood samples. We evaluated these new kits, as well as the Tempus Spin RNA Isolation kit, for the simultaneous recovery of both mRNA and miRNA with regard to yield, quality, integrity and representation from whole blood collected in Tempus Blood RNA tubes.

## Main text

### Materials and methods

#### Blood collection and RNA extraction

Phase one included 12, and phase two included four anonymous healthy volunteers recruited from Centers for Disease Control and Prevention’s voluntary blood donation program. The clinic collected peripheral blood through antecubital venipuncture directly into 3 ml Tempus Blood RNA tubes (Thermo Fisher), followed by immediate vigorous shaking of each tube for 10 s. Three tubes were collected from each participant for phase one, and four tubes for phase two. Samples were stored at room temperature for 2 h, then stored at − 20 °C until RNA extraction.

In phase one, we manually extracted total RNA to compare three different kits: two filter-based kits [Tempus Spin RNA Isolation kit (Thermo Fisher) and Norgen Preserved Blood RNA purification kit I (Norgen Biotek Corp)], and one magnetic bead-based kit [MagMax for stabilized blood tubes RNA isolation kit (Thermo Fisher)]. Frozen blood tubes were thawed prior to RNA extraction following the manufacturer’s protocol for each kit. Tempus Spin and Norgen protocols did not include a DNase treatment whereas the MagMax protocol included a DNase treatment. For the second phase, the manual MagMax protocol (with and without DNase treatment) was compared to semi-automated MagMax extraction (with and without DNase treatment) per the instructions of the MagMax Express 96 instrument (Thermo Fisher).

#### Determination of RNA yield and integrity

We determined RNA yield and purity based on 260/280 and 260/230 ratios with a NanoDrop ND-1000 spectrophotometer (Thermo Fisher). We determined RNA integrity number (RIN values range from 1 to 10, with higher values indicating less degradation) and the fraction of small RNAs (tRNA, small rRNA and miRNA) with the Agilent 2100 Bioanalyzer (Agilent Technologies, Santa Clara, CA) using the Eukaryote RNA 6000 Nano and the small RNA kits (Agilent). The small RNA analysis indicates the region enriched for miRNA.

#### Reverse transcription (RT)

Two RT reactions were prepared for each sample, one for mRNA and one for miRNA. For mRNA, 0.5 µg total RNA in a 20 µl reaction was transcribed using the PrimeScript™ RT reagents Kit with gDNA eraser (Takara Bio, Mountain View, CA) following manufacturer’s recommendations. After gDNA eraser treatment for 2 min at 42 °C, a 1 µl aliquot was removed for use as the no-RT control. The remainder of the reaction volume underwent the RT reaction at 37 °C for 15 min. For miRNA, 0.5 µg total RNA in a 25 µl reaction was transcribed using the All-in-One™ miRNA qRT-PCR Reagent Kit (GeneCopoeia, Rockville, MD) following manufacturer’s recommendations (37 °C for 1 h). The GeneCopoeia kit includes reagents for polyadenylation of mature miRNA followed by reverse transcription using modified oligo (dT) with a universal adaptor.

#### Quantitative PCR (qPCR)

We selected nine mRNA and nine miRNA targets based on their reported abundance in blood [5]. mRNA targets were β-actin (ACTB), matrix metallopeptidase 9 (MMPP), arginase 1 (ARG1), glyceraldehyde 3-phosphate dehydrogenase (GAPDH), interleukin-2 receptor alpha chain (CD25/IL2RA), hypoxanthine phosphoribosyltransferase 1 (HPRT1), forkhead box P3 (FOXP3), phosphoglycerate kinase 1 (PGK1), and peptidyl-prolyl cis–trans isomerase B (PPIB). miRNA targets were miR-16-5p, miR-30b-5p, miR-133a-3p, miR-155, miR-191, miR-103, miR-223, miR-26b and snRNA. qPCR was done in 96 well plates using LightCyler 480 (Roche Diagnostic Corporation, Indianapolis, IN) in SYBR Green mode with All-in-One™ qPCR Mix kit (GeneCopoeia). mRNA and miRNA targets were tested in duplicate, in 20 µl reaction with 2 µl and 2.5 µl of 1:20 dilution of respective cDNA products. We used validated primers supplied by GeneCopoeia for selected mRNA (All-in-One qPCR Primer) and miRNA (All-in-One miRNA Primer) targets. Each 96-well plate included positive and negative (no-RT and water controls) controls. For mRNA targets, the positive control was cDNA derived from Stratagene Human Reference total RNA (Agilent Technologies, Santa Clara, CA). For miRNA targets, the positive control was cDNA derived from a Universal Human miRNA Reference RNA (Agilent). Unless otherwise indicated, thermal cycling conditions were 1 cycle of enzyme activation (95 °C for 10 min), 40 cycles of amplification (95 °C 10 s, 60 °C 20 s, 72 °C 15 s (10 s for miRNA), and melting (95 °C 5 s, 65 °C 1 min, 95 °C continuous). The second derivative calculation was used to determine the fractional PCR cycle (Cq, quantitative cycle) for the relative quantification of mRNA and miRNA targets.

#### Data analysis

Total RNA yield, RNA purity (absorbance ratios), RNA integrity and small RNA yield were expressed as mean ± standard error of mean (SEM) for all samples processed with each kit, and comparisons used One-way ANOVA. Fold differences in the representation of mRNA or miRNA targets were calculated by 2^−ΔΔCq^ [6] where Cq value of a mRNA or miRNA target gene was normalized to geomean of two previously validated endogenous reference genes, PPIB and PGK1, for stabilized whole blood [7], and then expressed relative to the current Tempus extraction method. For example, fold difference in the representation of miR-16-5p by MagMax relative to Tempus will be calculated using the equation, 2^−((A−B)−(C−D))^ where A = Cq of miR-16-5p (MagMax), B = geomean Cq of PPIB and PGK1 (MagMax), C = Cq of miR-16-5p (Tempus) and D = geomean Cq of PPIB and PGK1 (Tempus). The resulting relative value assumes primers for both targets and reference genes amplify with nearly equal efficiencies. All samples were restricted to the same set of PCR conditions for targets and reference genes.

### Results and discussion

#### RNA yield, purity and integrity

RNA yields from all three protocols (Table 1) were within the ranges reported previously [2, 8], and within the range of manufacturers’ specifications (3–25 µg). While total RNA yield did vary by kit, the differences were small (range 8.34–12.04 µg). Similarly the mean 260/280 ratios differed statistically by kit, but were all close to two, indicating acceptable purity. While the significance of the 260/230 ratio is not well established, the mean 260/230 ratios were statistically significant, with only Norgen indicating a ratio close to two. The reason for the differences is unclear but high salt content in the elution buffer may play a role [9].

**Table 1:** Impact of extraction kits on total RNA yield, purity, integrity and small/miRNA yield

Characteristics	Tempus	Norgen	MagMax	p value
Total RNA yield (µg)	10.14 ± 0.73	12.04 ± 0.85	8.34 ± 0.76	0.008
Total RNA purity (260/280 ratio)	2.05 ± 0.01	2.09 ± 0.01	2.09 ± 0.01	0.027
Total RNA purity (260/230 ratio)	1.25 ± 0.05	1.98 ± 0.32	1.13 ± 0.06	< 0.0001
Total RNA integrity (RIN values)	8.70 ± 0.05	8.63 ± 0.05	6.68 ± 0.14	< 0.0001
Total RNA integrity (28S:18S ratio)	1.69 ± 0.04	1.54 ± 0.03	1.26 ± 0.06	< 0.0001
Small RNA yield (ng)	271.9 ± 34.2	344.8 ± 25.7	362.6 ± 24.8	Not significant
miRNA yield (ng)	24.7 ± 0.05	17.9 ± 1.6	39.3 ± 5.6	0.011
miRNA/Small RNA (%)	8.16 ± 1.6	5.17 ± 0.2	11.18 ± 1.8	0.014

All values given are mean ± standard error of mean

RIN values for individual RNA extracts with the Tempus and Norgen kits ranged from 8.4 to 9.0 whereas those with MagMax kit ranged from 5.9 to 7.5 (data not shown); the mean RIN for all samples with each kit reflect these differences (Table 1). While the MagMax RIN values are slightly below those of the Tempus and Norgen, they are within ranges reported by the manufacturer and others [2, 3, 8]. Generally, all three kits yielded RNA extracts with RIN values ≥ 7 which is considered satisfactory for gene expression analysis by methods like microarray, RT-PCR and RNA-seq. Furthermore, these slightly lower RIN values did not affect qPCR results (see next section) and appear not to be an important deciding factor for downstream microarray and RNA-Seq applications [10–12]. Since rRNA (28S:18S) ratios for all samples extracted with all three kits (except one with MagMax) were > 1.00, a cut-off for most gene expression assays, this ratio was not a differentiating feature for kit selection.

While there was only small differences among kits in their total RNA yields, perhaps reflecting their design for mRNA, they did differ in mean yields for miRNA as well as proportion of miRNA/small RNA (Table 1). The mean miRNA for MagMax extraction was 1.6–2.2-fold more than that for Tempus and Norgen kits. No significant correlation was observed between the RIN values and miRNA yield for any of the kits (data not shown).

#### Impact of kits on the representation of mRNA and miRNA

**Fig. 1:**
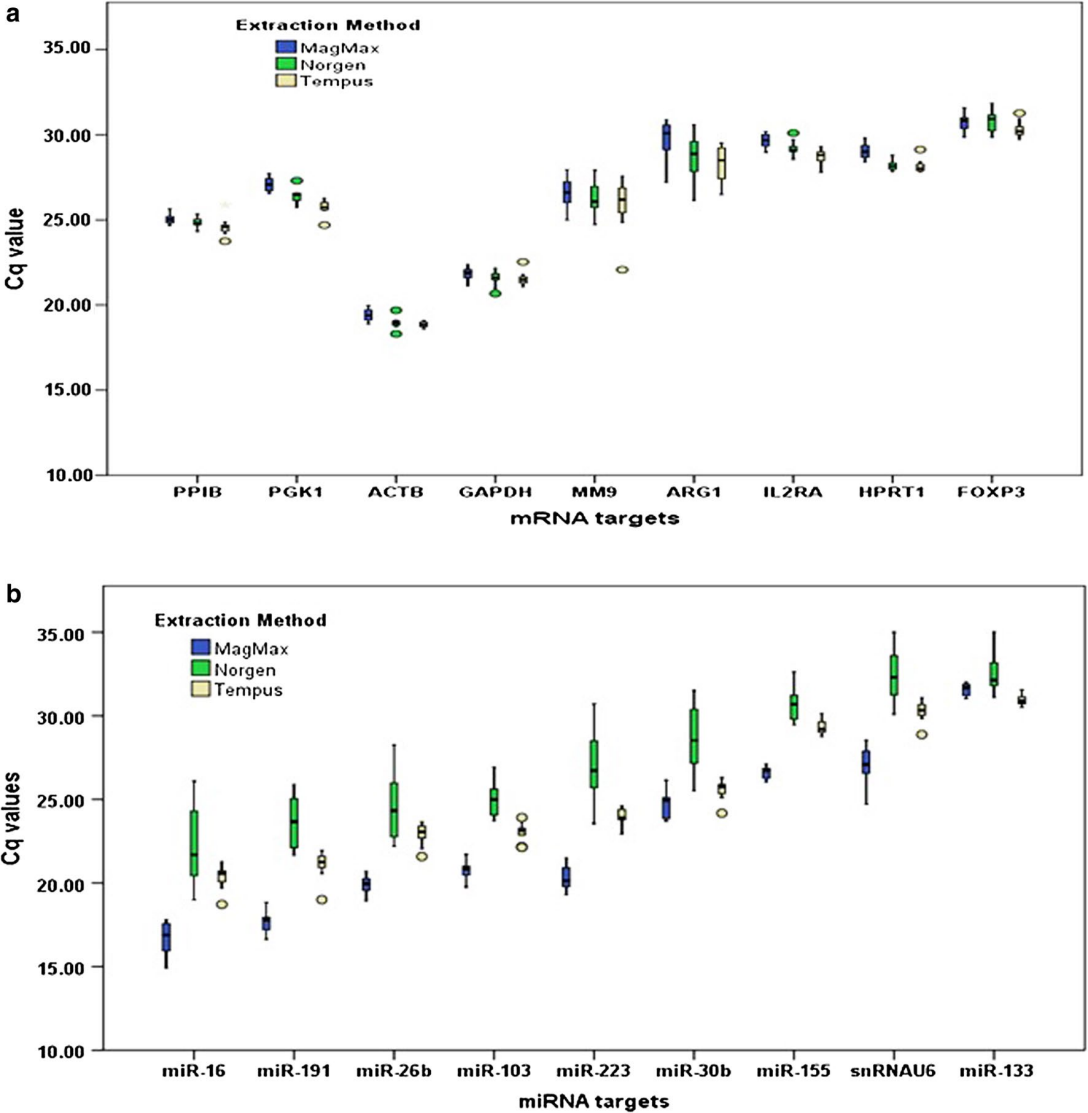
Impact of extraction kits on the representation of specific mRNA and miRNA targets. Values plotted in box-plots are raw Cq values. **a** mRNA representation is unaffected by the extraction kits. **b** miRNA representation affected by the extraction kits

The negative controls did not vary: all no-RT and water-controls were negative for GAPDH. The positive controls (cDNAs synthesized from human total RNA and human universal miRNA references) performed on different days throughout the study gave highly reproducible Cq values for each mRNA and miRNA target. Based on 2^−ΔΔCq^ analysis using raw Cq values for the selected target, mRNA representation differed by less than twofold between kits (Fig. 1a). However, miRNA representation varied by kit (Fig. 1b). Samples extracted with MagMax averaged a more than 12-fold enrichment of miRNA targets in comparison to Tempus, and samples extracted with Norgen averaged 3.3-fold less than miRNA targets compared to Tempus.

#### Manual vs automated extraction using MagMax

**Fig. 2:**
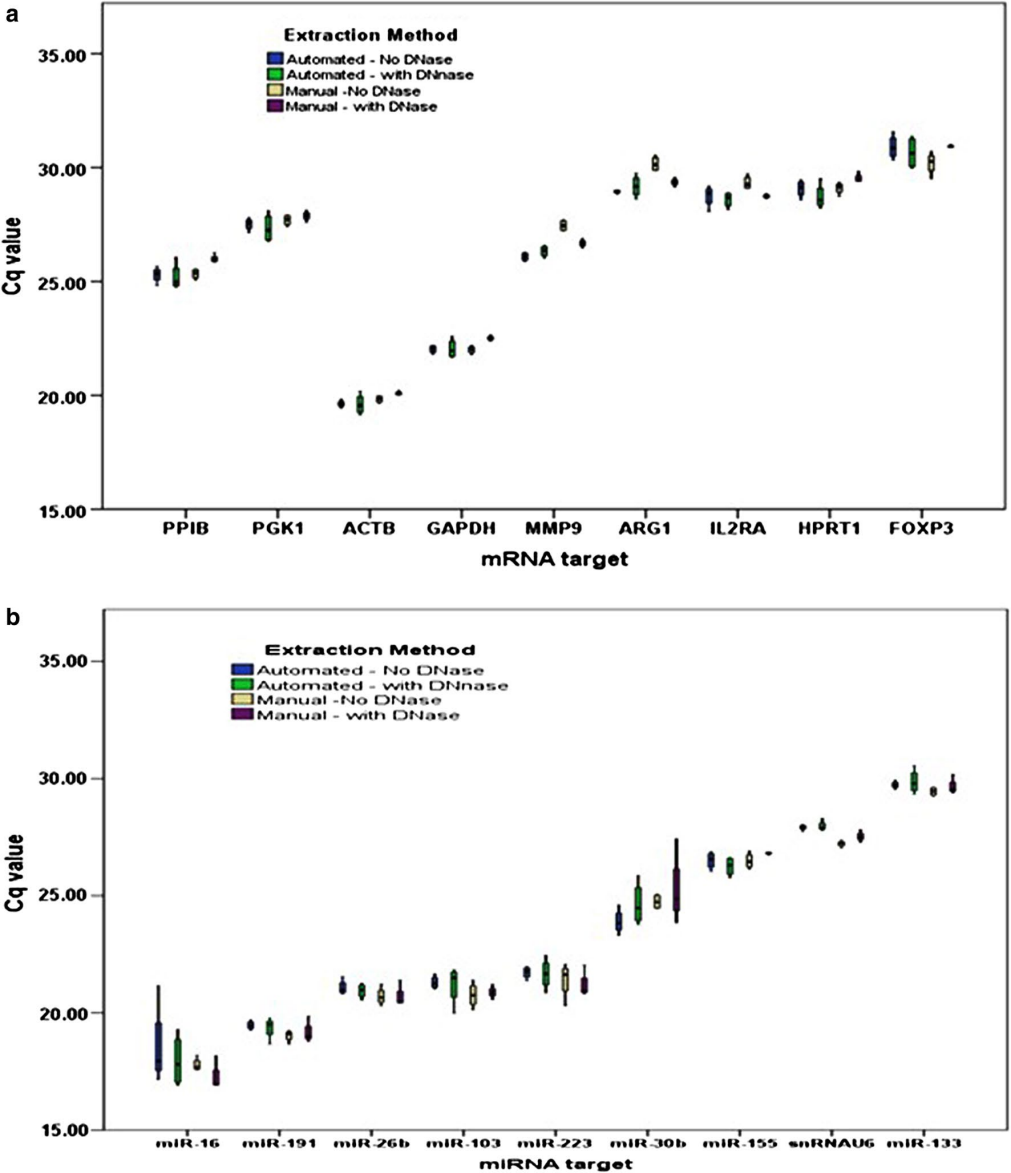
Automation and DNase treatment using MagMax kit. Values plotted in box-plots are raw Cq values. Both mRNA representation (**a**) and miRNA representation (**b**) remained unaffected by semi-automation or DNase treatment step

While MagMax RNA extraction has the advantage for miRNA retention, automation is desirable because the manual method is tedious and potentially prone to errors. We compared results achieved with an automated protocol to those with manual. Both methods, with or without DNase treatment, gave similar results on all parameters [total RNA yield, 260/280 ratio, 260/230 ratio, RNA integrity, small RNA and miRNA yield, mRNA representation (Fig. 2a), miRNA representation (Fig. 2b)].

A key finding from this study is that RNA extraction kits have little impact on representation of mRNA but show a marked impact on miRNA representation. For simultaneous recovery of both mRNA and miRNA from stabilized blood in Tempus tubes, the MagMax protocol is favored. Hantzsch et al. [2] reported a similar conclusion on the representation of mRNA and miRNA, although their supporting data is inconclusive. The raw Cq values appear similar between RNA extraction kits, calculation of fold differences with reference to a kit is not provided, and no specific kit is finally recommended for enrichment of miRNA from stabilized blood in Tempus tubes. On the other hand, a recent study reported that representation of both mRNA and miRNA isolated from stabilized blood in Tempus tubes was not affected by RNA isolation method [3], however, that study included Tempus Spin RNA Isolation kit, one that the manufacturer does not recommend for miRNA recovery. The Norgen kit is marketed as a new generation kit for simultaneous recovery of both mRNA and miRNA from whole blood stabilized in Tempus tubes, but our results do not support this, based on the lowest miRNA yield (Table 1) and poor representation of all miRNA targets (as indicated by the highest Cq values for all miRNA targets, Fig. 1b) in the RNA extracted with Norgen kit,

## Limitations

Comparison of three kits recommended for the extraction of total RNA from whole blood stabilized in Tempus blood tubes found the magnetic bead-based MagMax kit superior to the filter-based Norgen and Tempus extraction kits in terms of simultaneous recovery of both mRNA and miRNA with significant enrichment of the miRNA fraction. While our results favor the MagMax kit, the determination is based on only a snapshot of the complexity and representation of RNA in whole blood that a limited number of mRNA and miRNA targets provide. However we feel the guidance provided is useful because of judicial selection of targets that represent the spectrum of abundance and sizes of total RNA species for most applications.

## Abbreviations

mRNA: messenger RNA
miRNA: microRNA
RT: reverse transcription
qPCR: quantitative PCR
ACTB: β-actin
MMPP: matrix metallopeptidase 9
ARG1: arginase 1
GAPDH: glyceraldehyde 3-phosphate dehydrogenase
CD25/IL2RA: interleukin-2 receptor alpha chain
HPRT1: hypoxanthine phosphoribosyltransferase 1
FOXp3: forkhead box P3
PGK1: phosphoglycerate kinase 1
PPIB: peptidyl-prolyl cis–trans isomerase B
SEM: standard error of mean
Cq: quantitative cycle
